# Bone metastasis is associated with acquisition of mesenchymal phenotype and immune suppression in a model of spontaneous breast cancer metastasis

**DOI:** 10.1038/s41598-020-70788-3

**Published:** 2020-08-14

**Authors:** Lea Monteran, Nour Ershaid, Idan Sabah, Ibrahim Fahoum, Yael Zait, Ophir Shani, Noam Cohen, Anat Eldar-Boock, Ronit Satchi-Fainaro, Neta Erez

**Affiliations:** 1 Department of Pathology, Sackler Faculty of Medicine, Tel Aviv University, 69978 Tel Aviv, Israel; 2 Department of Pathology, Tel Aviv Sourasky Medical Center, Tel Aviv University, Tel Aviv, Israel; 3 Department of Physiology and Pharmacology, Sackler Faculty of Medicine, Tel Aviv University, 69978 Tel Aviv, Israel

**Keywords:** Cancer models, Cancer, Breast cancer, Cancer microenvironment, Cancer models

## Abstract

The most common site of breast cancer metastasis is the bone, occurring in approximately 70% of patients with advanced disease. Bone metastasis is associated with severe morbidities and high mortality. Therefore, deeper understanding of the mechanisms that enable bone-metastatic relapse are urgently needed. We report the establishment and characterization of a bone-seeking variant of breast cancer cells that spontaneously forms aggressive bone metastases following surgical resection of primary tumor. We characterized the modifications in the immune milieu during early and late stages of metastatic relapse and found that the formation of bone metastases is associated with systemic changes, as well as modifications of the bone microenvironment towards an immune suppressive milieu. Furthermore, we characterized the intrinsic changes in breast cancer cells that facilitate bone-tropism and found that they acquire mesenchymal and osteomimetic features. This model provides a clinically relevant platform to study the functional interactions between breast cancer cells and the bone microenvironment, in an effort to identify novel targets for intervention.

## Introduction

Mortality from breast cancer is almost exclusively a result of metastatic relapse to distant organs. The most common site of breast cancer metastasis is the bone, occurring in approximately 70% of patients with advanced disease. Bone metastases are typically incurable and 5-year survival rate of patients with bone-metastatic relapse drops to < 10%^1,2^. Most of breast cancer metastasis to bone is osteolytic (bone destructive)^3,4^ and encompass severe morbidities, including pathologic fractures, pain, and hypercalcemia^1,5,6^. Therefore, deeper understanding of the mechanisms that enable bone metastatic relapse is an urgent medical need, in order to inhibit or prevent this devastating pathology.

Metastasis is a complex multistep process^7^. In many tumor types, including breast cancer, there is a temporal lag between the arrival of disseminated tumor cells to distant organs and their acquisition of capabilities that allow organ colonization^8^. It is increasingly appreciated that the metastatic microenvironment is crucial in supporting metastases formation^9^, and that disseminated malignant cells must acquire the capability to reprogram stromal cells in their new microenvironment to support their growth and allow the formation of clinically relevant metastases^9,10^. Indeed, disseminated tumor cells in the bone marrow have been observed in more than 50% of breast cancer patient already at the time of diagnosis^11^, and yet only 20% of breast cancer patients will ultimately develop macrometastases, suggesting that further interactions with the bone microenvironment are crucial. Pre-metastatic preparation of secondary sites to facilitate subsequent tumor cell colonization has been described for multiple cancers^12^. Secreted factors and extracellular vesicles from tumor and stromal cells were reported to instigate a permissive pre-metastatic niche by influencing the recruitment and activation of immune cells^13–17^, and by modifying the composition of the extracellular matrix (ECM)^18–22^.

Organ-specific metastasis results from both tumor cell-intrinsic mechanisms and the metastatic microenvironment^23^. Each metastatic microenvironment exerts specific functions that support or oppose colonization by disseminated tumor cells^8,12^. Therefore, understanding the distinct organ-specific mechanisms that enable metastatic growth is of crucial importance. The bone is a unique microenvironment comprising a great variety of cell types, including bone remodeling stromal cells such as osteoblasts, osteoclasts and osteocytes that maintain bone homeostasis and integrity, mesenchymal stem cells (MSCs), and a rich variety of immune cells in the bone marrow (BM), which is the main site of hematopoiesis^1^. The interactions between tumor cells and the bone microenvironment underlie the ability of tumor cells to disrupt bone homeostasis, instigate bone degradation and form a hospitable metastatic niche^24–27^. Nevertheless, knowledge on the early mechanisms that facilitate spontaneous bone metastasis is limited by the fact that most studies rely on systemic (intravenous) or local (intra-tibial) injections of tumor cells, often using human cell lines in immunodeficient mice, giving rise to experimental metastases, which do not faithfully recapitulate the multistage process of metastasis^28–30^, and enable limited insight on the critical early stages of metastasis, and on the role of immune cells. Investigating metastasis in an immunocompetent context is essential, given the critical role of immunosuppression on tumor progression and metastasis, and the emerging importance of immunotherapies^31,32^.

Here we report the establishment and characterization of a breast cancer bone-metastasizing cell line following surgical removal of the primary tumor. In vivo selection gave rise to aggressive bone-seeking variants of breast cancer cells that spontaneously metastasize to bone. We characterized the changes in breast cancer cells associated with the formation of bone tropism and found that bone-tropic cells acquire mesenchymal characteristics, and that the formation of bone metastases is associated with modifications of the bone metastatic microenvironment towards an immune suppressive milieu, as well as systemic inflammation and immunosuppressive environment in spleen and in blood. This system provides a clinically relevant experimental platform to study the early interactions between disseminated tumor cells and the bone microenvironment and enables future characterization of the dynamic changes in the bone metastatic niche.

## Results

### In vivo selection for bone-metastasizing cells generates a spontaneous model of breast cancer bone metastasis

**Figure 1:**
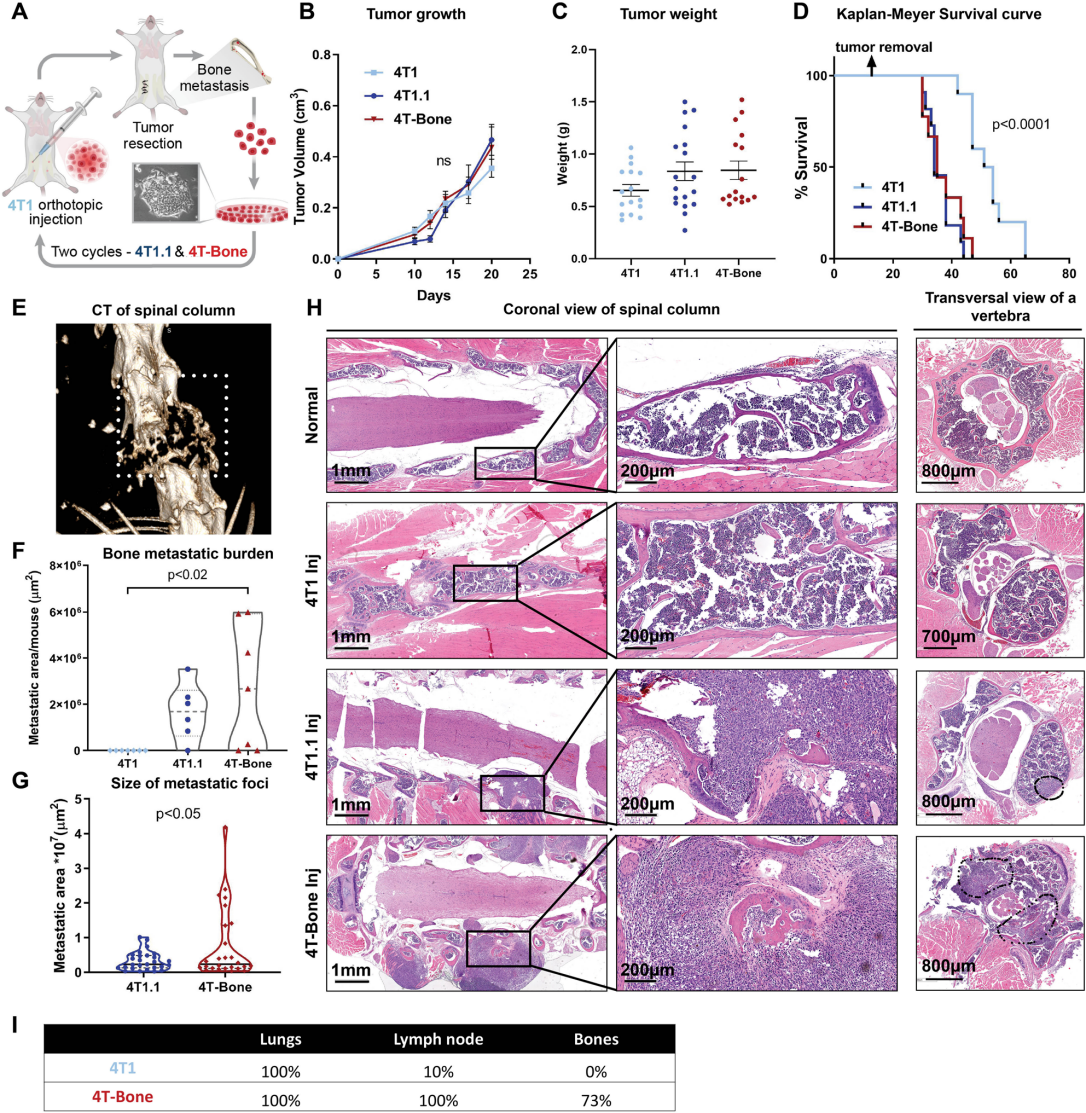
Generation of a mouse model of spontaneous breast cancer bone metastasis by in vivo selection. (**A**) Schematic summary: a model of spontaneous breast cancer bone metastasis generated by in vivo selection. Two cycles were performed, creating 4T1.1 and 4T-Bone clones. (**B**) Tumor growth curve of the different clones; n = 7 mice per group. Error bars represent SEM. (**C**) Tumor weight of the resected primary tumors. Error bars represent SEM. Kruskal–Wallis test. (**D**) Kaplan–Meyer survival curve of mice injected with the three clones; 4T1: n = 10, 4T1.1: n = 11, 4T-Bone: n = 9. Log-rank (Kruskal–Wox) test. (**E**) Representative μCT image of spinal metastasis. (**F**) Bone metastasis burden of the different clones, Kruskal–Wallis test. (**G**) Quantification of bone metastatic area in mice injected with bone-tropic cells. Welch's t-test. n = 7 (4T1), n = 6 (4T1.1), n = 7 (4T-Bone) for (F&G). (**H**) Representative images of H&E sections of bone metastasis. Left, middle: coronal view of spinal column w/wo bone metastasis. Right: transversal view of a vertebra. (**I**) Metastatic incidence in lungs, lymph nodes and bones in 4T1 or 4T-Bone injected mice at day 40. n = 6 per group.

In order to enable investigation of the metastatic microenvironment in spontaneous bone metastases, we generated a bone seeking variant of the murine triple negative breast cancer cell line 4T1 by in vivo passaging in immune competent syngeneic mice, as previously described^33^. 4T1 cells were orthotopically inoculated to the fourth mammary gland of 6 weeks old BALB/c female mice. To mimic the clinical setting of metastatic relapse, we surgically resected the primary mammary tumors. Surgeries were performed when the average size of primary tumors was 0.5 cm^3^, typically around day 16–18. Following tumor resection, mice were monitored for signs of morbidity (e.g. difficulty in breathing, movement, paraplaysia) and euthanized upon metastatic relapse. Spontaneous bone metastases formed in < 10% of mice. We isolated and cultured cells from a spontaneously occurring bone metastases and designated them 4T1.1. These cells were re-injected to mammary glands for another cycle of in vivo selection and culture. These bone-metastasizing cells were designated 4T-Bone (Fig. 1A). Analysis of the primary tumor growth of the bone-seeking variants compared with the parental cells revealed a trend towards enhanced growth in the bone-seeking variants, manifested by enhanced tumor volume and weight, but the differences were not statistically significant (Fig. 1B,C). However, we compared the aggressiveness of the different clones by analyzing the survival of injected mice after removal of the primary tumors, and found that mice injected with the bone-metastasizing clones had significantly reduced survival as compared with the parental cell line (Fig. 1D).

We next compared the bone metastatic burden of mice inoculated with 4T1, 4T1.1 or 4T-Bone at end-stage. End-stage was assessed during calibration of the model by detailed histopathological analysis, and determined to be around day 40, in which most of 4T-Bone injected mice had signs of morbidity and evident bone macrometastases (Fig. 1E,H). None of the 4T1-injected mice had developed bone metastases at the determined end-stage, whereas 63% and 70% of 4T1.1 and 4T-Bone-injected mice, respectively, developed bone macrometastases, as confirmed by intravital CT imaging (Fig. 1E) and histopathological analysis of bone tissue sections from spinal column, legs, ribs and sternum (Fig. 1H). Of note, since tumor cells were not labeled, we cannot rule out the presence of disseminated tumor cells that did not form macrometastases. Quantification of bone metastases revealed that while there was no significant difference in metastatic incidence and burden between 4T1.1 and 4T-Bone (Fig. 1F), bone metastases in the bones of mice injected with 4T-Bone were significantly larger than bone metastases in 4T1.1-injected mice (Fig. 1G). Moreover, we analyzed the incidence of lung metastasis and lymph node metastasis in mice injected with 4T1 or with 4T-Bone. While all mice had lung metastasis, mice injected with 4T-Bone had a striking increase in lymph node metastasis, consistent with previous studies^34^ (Fig. 1I). Thus, in vivo selection generated aggressive spontaneously bone-metastasizing breast cancer cells.

### Bone metastases are associated with an immunosuppressive microenvironment

**Figure 2:**
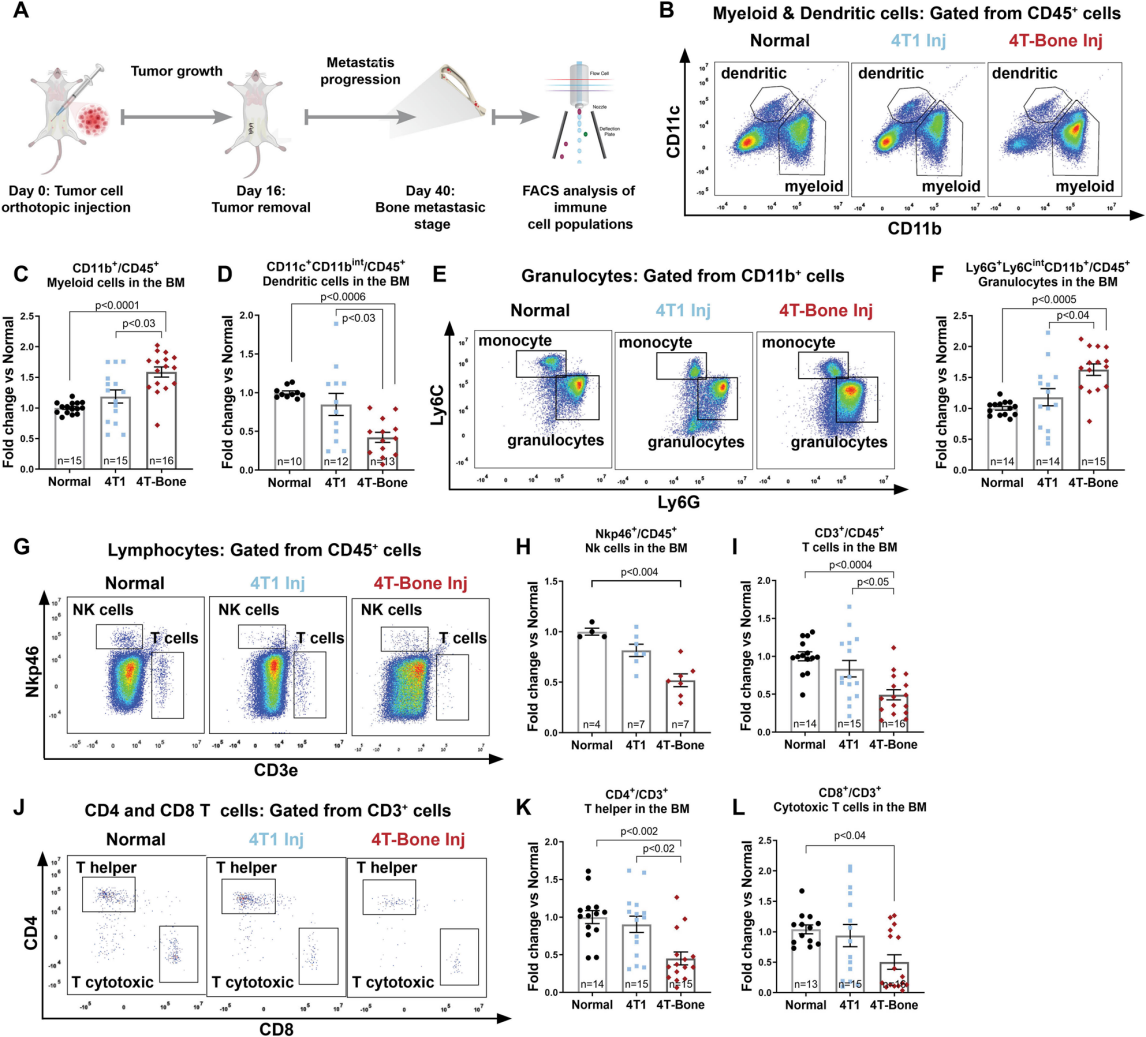
Bone metastases are associated with an immunosuppressive milieu. (**A**) Scheme of experimental design. Bone marrow of mice with bone metastases was flushed and the immune landscape was analyzed by flow cytometry. (**B**–**D**) Representative FACS gating and quantification of the total myeloid and dendritic compartment (CD45^+^CD11b^+^/CD11c^+^) for each group (normal, 4T1 or 4T-Bone injected mice). (**E**, **F**) Representative FACS gating and quantification of granulocytes (CD11b^+^Ly6G^+^Ly6C^int^). (**G**–**I**) Representative FACS gating and quantification of T and NK cells within CD45^+^ cells. (**J**–**L**) Representative gating and quantification of T helper cells (CD3^+^CD4^+^) and cytotoxic T cells (CD3^+^CD8^+^). Number of mice per each group is specified in graphs. Data are presented as % of CD45 cells, normalized to the average of the normal group. Error bars represent SEM. *p* < 0.05; Kruskal–Wallis multiple comparison test.

Modeling spontaneous bone metastasis in immunocompetent mice provides a platform to investigate the immune milieu and the dynamic changes in the immune microenvironment during the multi-stage process of metastases formation. To analyze these changes, we utilized flow cytometry to assess the main immune cell populations within the bone marrow (BM) of normal mice as compared with BM at end-stage from mice injected with 4T1 or 4T-Bone (Fig. 2A). At this time point, most of the 4T-Bone-injected mice had bone metastases. Analysis of immune cell populations revealed a significant increase in myeloid cells (Fig. 2B,C). In particular, there was a striking increase in granulocytes (Fig. 2E,F) in 4T-Bone-injected mice, compared to controls. Moreover, we found a decrease in dendritic cells (Fig. 2B,D) and in NK cells (Fig. 2G,H). Analysis of lymphocytic cells revealed a decrease in the populations of T lymphocytes (Fig. 2G,I). Specifically, CD4^+^ and CD8^+^ T cells were decreased in the BM of 4T-Bone injected mice (Fig. 2J–L). These findings suggest that the formation of bone metastases may be associated with immune suppression in the BM. Interestingly, the immune microenvironment in mice inoculated with the parental 4T1 cells, was more similar to that of normal mice than to 4T-Bone injected mice, suggesting that a modified immune microenvironment is required to enable successful metastatic bone colonization.

**Figure 3:**
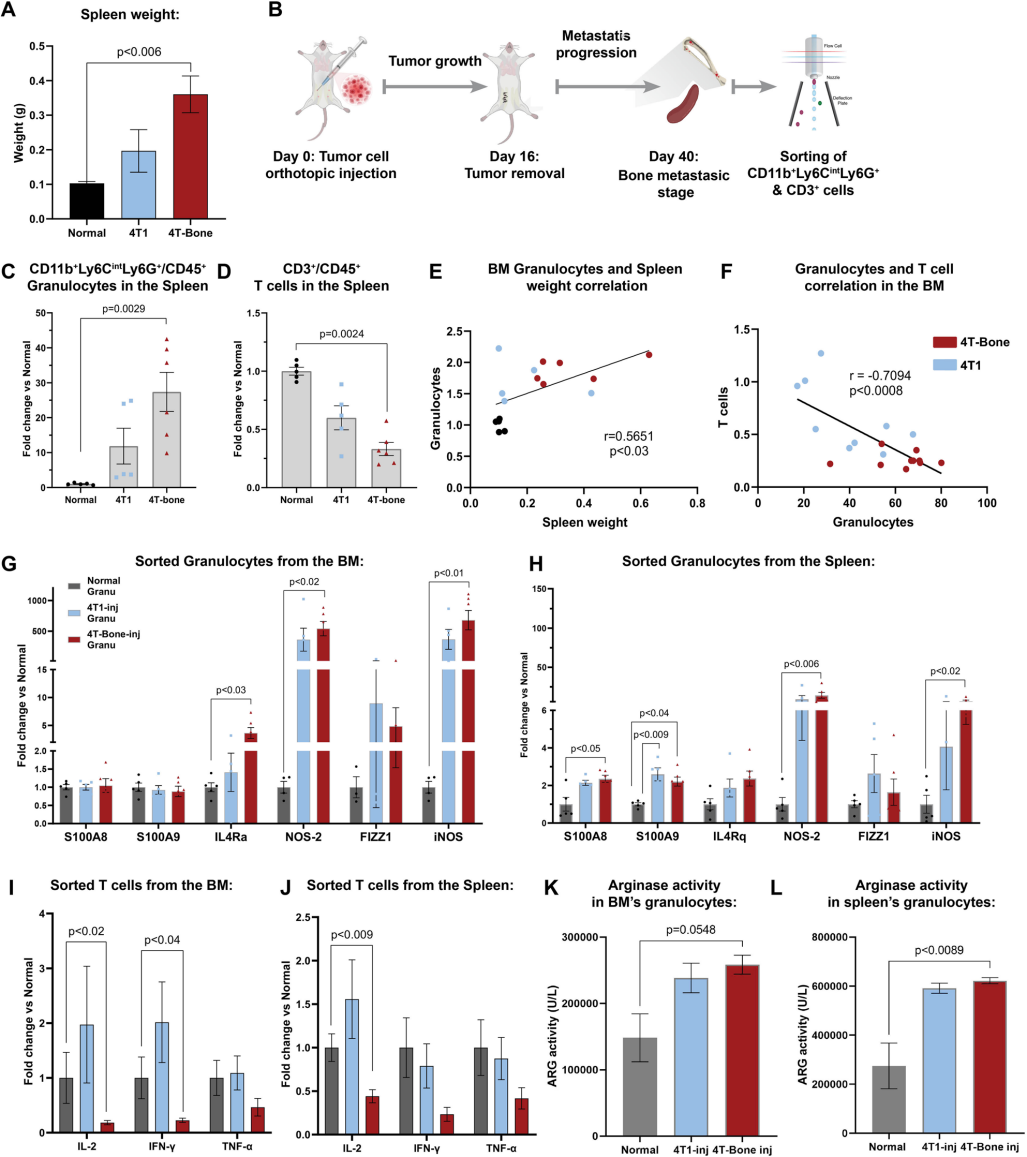
Granulocytes and T cells in BM and spleen comprise an immunosuppressive phenotype. (**A**) Spleen weight at day 40. Error bars represent SEM of biological repeats. n = 5–6. (**B**) Scheme of experimental design. Granulocytes (CD45^+^CD11b^+^Ly6C^int^Ly6G^+^) and T cells (CD45^+^CD3^+^) were analyzed and isolated. This scheme was designed by using graphical elements from BioRender. (**C**, **D**) Quantification of granulocytes and T cell population in the spleen of normal, 4T1-injected or 4T-Bone-injected mice. Error bars represent SEM of biological repeats. One way ANOVA was performed. n = 5–6. (**E**) Correlation between spleen weight and the abundance of granulocytes in the BM of 4T1 (n = 9) and 4T-Bone (n = 10) injected mice. (**F**) Correlation between Granulocytes and T cells in the BM of 4T1 (n = 9) and 4T-Bone (n = 10) injected mice. (**E**, **F**) Pearson correlation was performed. (**G**) qPCR analysis of the expression of immunosuppressive signature expressed by granulocytes isolated from the BM (**G**) and the spleen (**H**) of normal/4T1/4T-Bone-injected mice at day 40. (**G**, **H**) Error bars represent SEM of biological repeats. One-way ANOVA was performed. n = 5. (**I**) qPCR analysis of the expression of activation markers in T cells isolated from the BM (**I**) and the spleen (**J**) of normal/4T1/4T-Bone-injected mice at day 40. (**I**, **J**) Mann–Whitney test was perfomed. Error bars represent SEM of biological repeats. n = 5. (**K**, **L**) Arginase activity in sorted granulocytes from the BM (**K**) and spleen (**L**), One-way ANOVA was performed. Error bars represent SEM of biological repeats. n = 5.

Intrigued by these findings we set out to further investigate the changes in immune status in mice injected with 4T1 or 4T-Bone. Since the spleen is a major site of extramedullary hematopoiesis and was shown to be an origin of MDSCs in cancer^35^, we focused our further analyses on both BM and spleen. Interestingly, we observed significant splenomegaly in mice injected in 4T-Bone (Fig. 3A), suggesting that immune modulation during the formation of bone metastases is systemic. Based on our findings showing changes in granulocytes and T cells in the BM of mice with bone metastases, we next focused on these cell populations in the spleen and BM of injected mice (Fig. 3B). Analysis of cell populations revealed a striking increase in granulocytes (Fig. 3C) and decrease in T cells (Fig. 3D) in the spleens of mice injected with 4T-Bone, similarly to what we found in the BM (Fig. 2). Moreover, the increase in BM granulocytes correlated with the increase in spleen weight (Fig. 3E), and was also highly correlated with the decrease in T cells (Fig. 3F), supporting the hypothesis that the enhanced granulocyte population may be immunosuppressive. We therefore asked whether the modifications in the numbers of granulocytes and T cells is also associated with changes in the expression and function of known immunosuppressive factors. To that end, we isolated granulocytes and T cells from the BM and spleen of injected mice and analyzed their gene expression by qRT-PCR. We found that granulocytes from BM or spleen of mice injected with 4T1 or 4T-Bone upregulated an MDSC-like gene signature including IL-4Ra, NOS-2, FIZZ1 and iNOS (Fig. 3G,H). Moreover, analysis of T cells from mice injected with 4T-Bone (but not with 4T1) revealed downregulation of known T cell activation markers including IL-2, IFNγ and TNFα (Fig. 3I,J). Functionally, analysis of arginase activity in granulocytes from BM and spleen revealed enhanced activity in mice injected with 4T-Bone (Fig. 3K,L), indicative of myeloid suppressive function that disrupts T cell-mediated killing. Taken together, these results imply that formation of bone metastasis is associated not only with accumulation of granulocytes and decreased T cells, but also with acquisition of an immunosuppressive phenotype.

**Figure 4:**
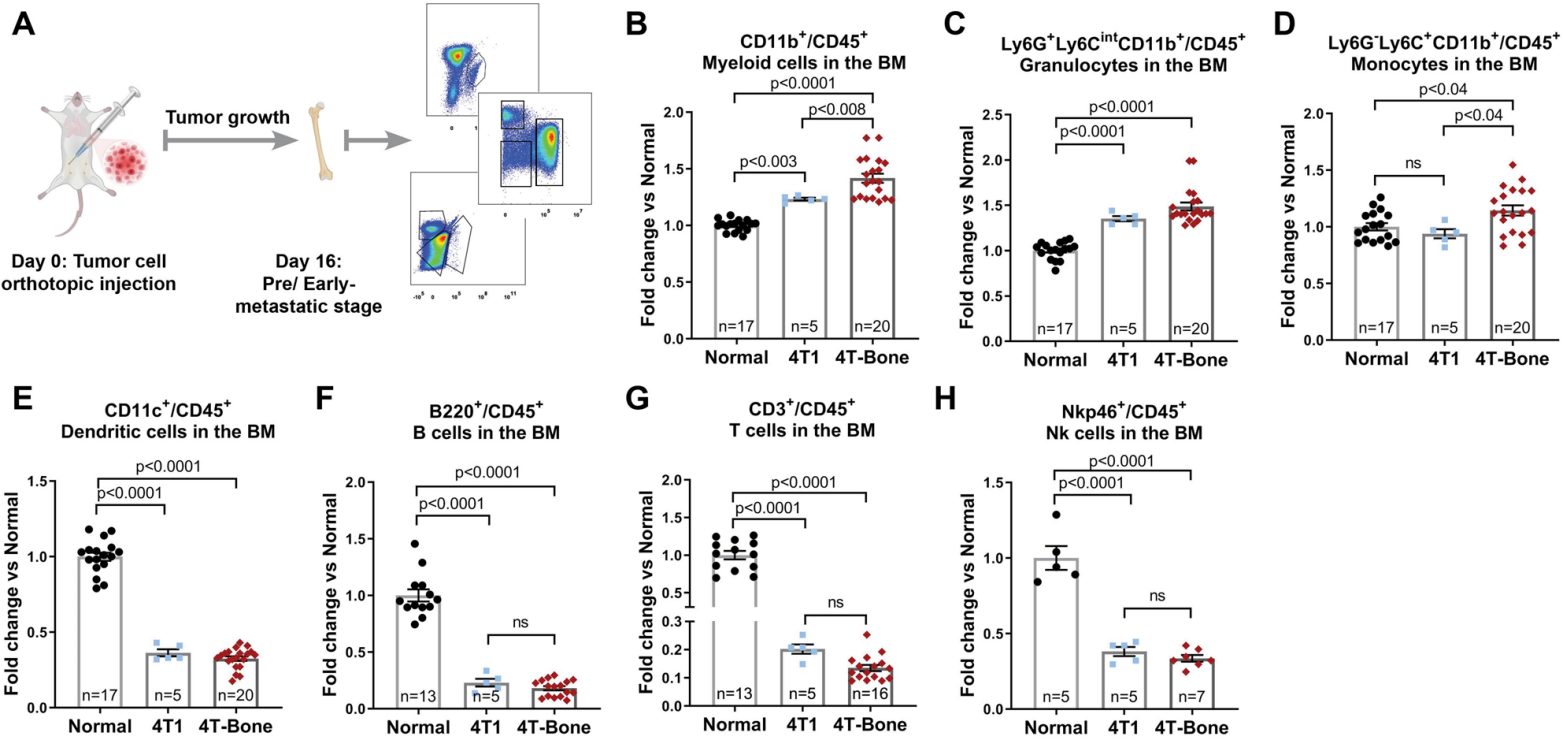
Immunosuppression in the BM precedes bone metastasis. (**A**) Scheme of the experimental design of early/pre-metastatic stage (day 16) analysis. (**B**) Quantification of the CD45^+^CD11b^+^CD11c^−^ total myeloid cell population. (**C**) Quantification of CD45^+^CD11c^−^CD11b^+^Ly6G^+^Ly6C^int^ granulocytes. (**D**) Quantification of CD45^+^CD11c^−^CD11b^+^Ly6G^−^Ly6C^+^ monocytes. (**E**) Quantification of CD45^+^CD11b^−^CD11c^+^ dendritic cells. (**F**) Quantification of CD45^+^CD3^−^B220^+^ B cells. (**G**) Quantification of CD45^+^B220^−^CD3^+^ T cells. (**H**) Quantification of CD45^+^B220^−^CD3^−^Nkp46^+^ NK cells. Number of mice per each group is specified in graphs. Data are presented as % of CD45, normalized to the average of normal group. Error bars represent SEM. *p* < 0.05; Kruskal–Wallis multiple comparison test.

Since modifications of the bone metastatic niche were shown to precede the formation of metastases^5,36^, we next asked whether the observed changes in the immune milieu of mice inoculated with bone-metastasizing cells are already evident at earlier stages of metastases formation. To that end, we analyzed immune cell populations in BM of mice at pre/early-metastatic stage, before the formation of bone macrometastases (Fig. 4A). Analyses at this pre/early-metastatic stage revealed striking changes in the bone immune milieu of mice that were injected with 4T1 or with 4T-bone: myeloid, granulocytic and monocytic cells were significantly elevated (Fig. 4B–D), while dendritic cells, NK cells and lymphocytes were very strongly suppressed (Fig. 4E–H). Surprisingly, the changes in immune cell populations at the pre/early-metastatic stage were even greater than those detected in metastases-bearing bones (Figs. 2, 4). Moreover, the increase in Ly6C^+^Ly6G^−^ monocytes in BM of 4T-Bone mice was evident only at the pre/early-metastatic stage, but not in bone metastases (Fig. 4 and data not shown). Interestingly, BM of mice inoculated with 4T1 cells, not capable of achieving bone metastatic relapse, also exhibited immunosuppressive changes that in most cell populations analyzed were similar to those in mice inoculated with 4T-bone. These findings suggest that pre/early-metastatic changes in the BM immune cell composition may be a result of systemic signaling from the primary tumor, prior to its resection, and that these changes are not sufficient to drive bone metastatic relapse.

### Early metastatic niche formation is associated with systemic inflammation and immunosuppression

**Figure 5:**
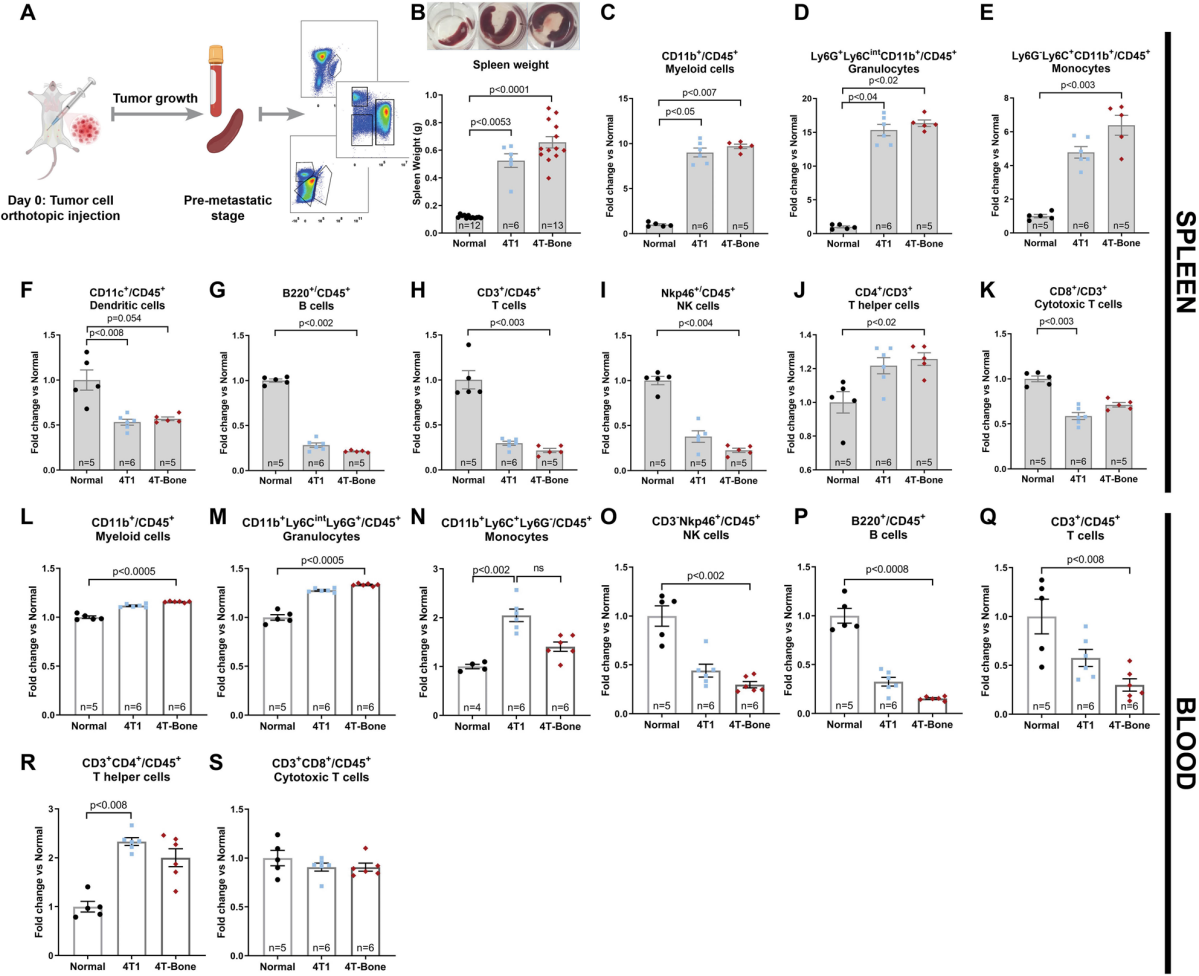
Breast cancer metastasis is associated with systemic inflammation and immunosuppression. (**A**) Scheme of the experimental design. This scheme was designed by using graphical elements from BioRender. (**B**) Representative images of spleens of normal, 4T1-injected and 4T-Bone-injected mice at pre-met stage. (**C**) Spleen weight showing severe splenomegaly in 4T1 and 4T-Bone injected mice. (**C**–**K**) FACS analysis results of major immune cell populations in spleens. (**C**) CD45^+^CD11b^+^CD11c^−^ total myeloid cell population. (**D**) CD45^+^CD11c^−^CD11b^+^Ly6G^+^Ly6C^int^ granulocytes. (**E**) CD45^+^CD11c^−^CD11b^+^Ly6G^−^Ly6C^+^ monocytes. (**F**) CD45^+^CD11b^−^CD11c^+^ dendritic cells. (**G**) CD45^+^CD3^−^B220^+^ B cells. (**H**) CD45^+^B220^−^CD3^+^ T cells. (**I**) CD45^+^B220^−^CD3^−^Nkp46^+^ NK cells. (**J**) CD45^+^B220^−^CD3^+^CD4^+^ T helper cells. (**K**) CD45^+^B220^−^CD3^+^CD8^+^ cytotoxic T cells. (**L**–**S**) FACS analysis results of circulating immune cells in the blood of normal, 4T1-injected and 4T-Bone-injected mice. Number of mice per each group is specified in graphs. Data are presented as % of CD45, normalized to the average of normal group. Error bars represent SEM. *p* < 0.05; Kruskal–Wallis multiple comparison test.

Based on these findings, we next assessed whether the early/pre-metastatic stages that precede bone metastases are associated with systemic inflammation, induced by the primary tumor. To that end, we profiled immune cell populations in the blood and the spleen (Fig. 5A).

Initial macroscopic examination of spleens revealed severe splenomegaly in mice injected with 4T1 or with 4T-Bone; up to two fold compared to the weight of spleens from metastases-bearing mice in the 4T-Bone group (Figs. 3A, 5B), indicating large production of leukocytes. Indeed, when we performed flow cytometry analysis we found a striking increase in myeloid populations, including monocytes and granulocytes (Fig. 5C–E), accompanied by a decrease in dendritic cells, B cells, T cells and NK cells (Fig. 5F–I), suggesting a dysfunction in normal immunity^37,38^. Moreover, when we analyzed specific T cell populations, we found an increase in T helper cells (CD3^+^CD4^+^) and a decrease in cytotoxic T cells (CD3^+^CD8^+^) suggesting systemic immune suppression (Fig. 5J,K).

We next analyzed the immune cells in the circulation of tumor cell-injected mice, compared with normal mice. The results recapitulated the observations in spleens: mice injected with either 4T1 or with 4T-Bone exhibited elevated levels of total myeloid cells and of granulocytes, and decreased levels of NK cells and lymphocytes (Fig. 5L–S), indicating systemic immune suppression. Notably, systemic instigation of an immunosuppressive microenvironment was also evident in pre/early-metastatic lungs (Supplementary Fig. 1). Thus, early stages of breast cancer metastasis are characterized by systemic changes in immune cells.

### Bone metastasizing breast cancer cells acquire a mesenchymal phenotype

**Figure 6:**
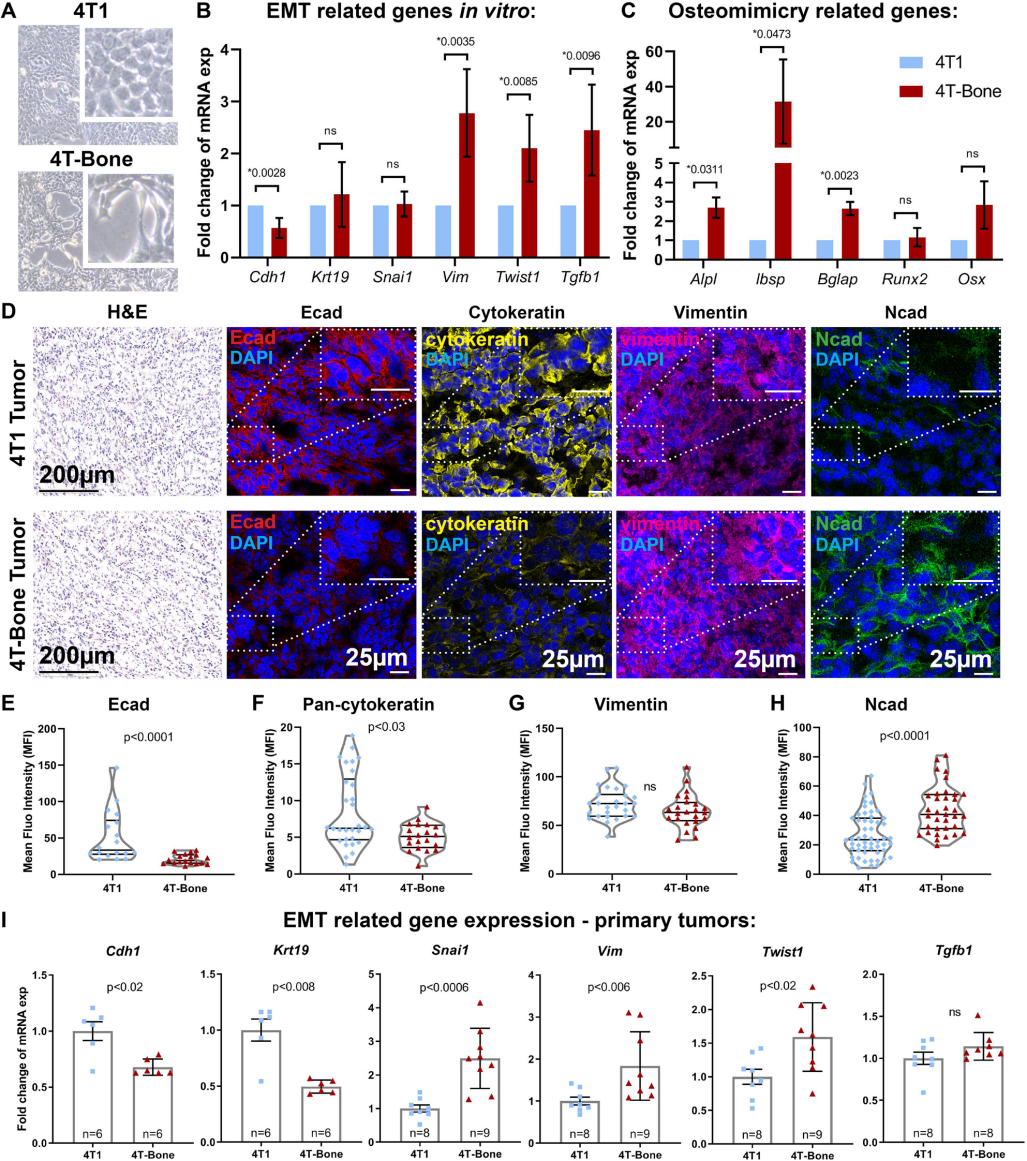
Bone metastasizing breast cancer cells acquire a mesenchymal phenotype. (**A**) Representative bright field images of in vitro cultured 4T1 and 4T-Bone cells, ×20 Magnification. Inset depicts an enlarged field. (**B**, **C**) qPCR analysis of the expression of EMT (**B**) and osteomimicry (**C**) related genes from 4T1 and 4T-Bone cell lines in vitro. Results presented are average of three biological repeats, normalized to 4T1 group. Paired t-test; *p < 0.05. Error bars represent SD of biological repeats. (**D**) Representative images of 4T1 and 4T-Bone breast cancer tissues showing (from left to right): H&E staining, scale bar-200 μm; immunofluorescence staining of Ecad (red); pan-cytokeratin (yellow); vimentin (pink); Ncad (green), nuclei were stained with DAPI (blue), scale bars-25 μm. (**E**–**H**) Quantification of IHC staining showed in (**D**), each dot represents a different field of view from multiple slides; n = 3 mice per group. Mean fluorescent intensity (MFI) was quantified using ImageJ and normalized to secondary antibody-only controls. Mann–Whitney test; *p < 0.05. (**I**) qRT-PCR analysis of EMT-related gene expression in primary 4T1 and 4T-Bone breast tumors. Results are presented as fold change of the average expression in 4T1 tumors. Each dot represents a different mouse. n = 6–9 mice, as indicated in graphs; Error bars represent SEM. *p < 0.05; Mann–Whitney test.

Both the parental 4T1 cells, which rarely form bone metastases, and the 4T-Bone metastasizing cells induced comparable systemic and BM changes in immune cell populations at early/pre-metastatic stage, but only 4T-Bone cells were capable of colonizing the bone. This led us to hypothesize that additional mechanisms are underlying the enhanced bone-metastasizing capacity of 4T-Bone cells. To address this hypothesis, we next analyzed intrinsic differences between 4T1 and 4T-Bone cells. Initial analysis of cell morphology indicated that bone metastasizing cells exhibited a more elongated cell morphology phenotype, reminiscent of mesenchymal cells (Fig. 6A). We therefore hypothesized that bone metastasizing capacity may be associated with activation of an epithelial-to-mesenchymal (EMT) program in tumor cells.

Epithelial-to-mesenchymal transition (EMT) is known to be associated with activation of transcriptional programs that enhance invasive and metastatic capacity in tumor cells^39^. A shift toward a more mesenchymal state was shown to modify the expression of adhesion molecules, support cell migration and invasion, and allow the dissociation of carcinoma cells from the primary tumor site^40^. We therefore analyzed the expression of genes associated with EMT in 4T1 as compared with 4T-Bone cells in vitro. Analysis of the results revealed that the expression of E-cadherin, an epithelial cell marker was significantly downregulated in 4T-Bone cells, whereas the expression of vimentin and TGFβ, associated with a mesenchymal phenotype was upregulated. Moreover, the expression of TWIST, a known EMT transcriptional regulator^39^ was also upregulated in 4T-Bone cells (Fig. 6B). Thus, acquisition of bone-metastasizing traits is associated with activation of an EMT gene expression program.

Notably, bone metastasizing tumor cells (e.g. breast and prostate cancer cells) were previously shown to exhibit a phenotype of "osteomimicry", in which tumor cells upregulate factors that are normally expressed in the bone microenvironment and are important for physiologic bone functions, including bone mineralization and remodeling^41,42^. We therefore analyzed the expression of bone-associated genes in 4T1 versus 4T-Bone cells and found that the bone tropic variant (4T-Bone) had significantly higher expression level of the bone physiology-related genes Alkaline phosphatase (ALP), Bone sialoprotein (BSP), Osteocalcin (OCN, BGLAP) and Osterix (Fig. 6C).

To assess whether the EMT-related changes between the parental cells and bone-metastasizing cells were operative in vivo, we analyzed tissue sections from primary tumors of mice injected with 4T1 or 4T-Bone. Immunostaining of EMT markers a striking downregulation of epithelial-like markers including E-cadherin and cytokeratins in 4T-Bone tumors and an upregulation in the expression of the mesenchymal marker N-Cadherin (Fig. 6D–H). Of note, in this analysis there was no significant change in vimentin, possibly reflecting its expression in stromal cells in the tumor microenvironment. Moreover, gene expression analysis of primary tumors confirmed the downregulation of epithelial genes (E-cad, keratin19), and upregulation of mesenchymal genes (vimentin, TGFβ), and upregulation of the EMT-associated transcription factors SNAI-1 and TWIST in vivo (Fig. 6I). Taken together, these findings suggest that acquisition of bone-metastatic capacity is associated with activation of an EMT program at the primary tumor.

In summary, our findings present a model of spontaneous bone metastasis, and suggest that reprogramming towards a more mesenchymal phenotype may increase the bone-metastasizing capacity of breast cancer cells.

## Discussion

We established a model of spontaneous breast cancer metastasis to bone by in vivo selection for a bone-tropic cell line (designated 4T-Bone) following removal of the primary mammary tumor, and characterized the changes in bone-seeking cancer cells and in the bone immune microenvironment.

It has become clear that the interactions of tumor cells with the bone microenvironment play a crucial role in enabling bone metastasis. Previous studies demonstrated the importance of bone-tropic gene expression in tumor cells^43^, and of reciprocal signaling between breast cancer cells and stromal cells in the bone that support osteolytic bone colonization^26,27,36,44^. However, detailed understanding of the early changes in the immune bone metastatic niche were hindered by the paucity in clinically relevant models that recapitulate the multistage process of metastasis in immune competent hosts^29^.

The triple-negative breast cancer cell line 4T1 was previously used to select for bone-tropic cells, giving rise to variants that were designated 4T1.2 and 4T1.3^33,45,46^. However, characterization of these cell lines in respect to the bone microenvironment and modulations of the immune milieu during the formation of spontaneous bone metastases is still incomplete.

We utilized the 4T-Bone variant to characterize the changes in the immune landscape during bone metastases formation and found that bone metastases are associated with an increase in myeloid cells and granulocytes and a decrease in T cells, dendritic cells and NK cells, suggesting that bone metastasis are characterized by immune suppression. Interestingly, we found that these changes preceded the formation of bone metastases and were evident already at early/pre-metastatic stages, as well as systemically in the blood and in spleen. Notably, the differences between 4T-Bone and the parental cell line were more significant in bone metastases compared with early/pre-metastatic stages, suggesting that acquisition of immunosuppressive capacity has a functional role in endowing cells with bone metastatic fitness. This observation is in agreement with previous findings with the 4T1.2 variant, showing that bone-tropic cells suppress the expression of type I IFN signaling^47^ and induce myeloid-derived suppressor cells (MDSCs)^48^, resulting in escape from immune surveillance in the bone. Interestingly, transcriptome profiling of patient-matched pairs of primary breast cancer and bone metastases indicated that bone metastatic tumors had reduced numbers of CD8^+^ T cells, regulatory T cells and dendritic cells, and elevated levels of M2-like macrophages^49^. Our findings imply that while systemic changes and immune modulation in the bone microenvironment occur also in primary breast cancer, acquisition of bone-metastatic capacity is associated with significant immunosuppressive changes in the bone.

In addition to elucidating the changes in the bone microenvironment, we analyzed the intrinsic differences between 4T1 and 4T-Bone. We found that bone-tropic cells activated an epithelial-mesenchymal transition gene expression program, combined with upregulation of known EMT transcription factors. The importance of EMT in promoting tumor cell migration and invasion is well known^39,50^. Our data suggest that EMT plays a role in the increased capacity of 4T-Bone cells to metastasize to the bones compared with the parental cells. However, EMT is not sufficient by itself to facilitate spontaneous breast cancer metastasis: the non-metastatic variant of the 4T1 cell line, 67NR was shown to express high levels of vimentin and N-Cadherin, and yet it is not invasive or metastatic^51,52^. Thus, other mechanisms that are non-cell intrinsic are required to support metastatic capability. Importantly, EMT was shown to be associated with immunological status in breast tumors, thus linking these two mechanisms^53,54^. Our findings that immune changes were evident also in 4T1 injected mice, that did not form bone metastases, suggest that dissemination capacity and the capacity to colonize the bone are distinct. This is in agreement with studies showing that disseminated cancer cells are found in the bone marrow of patients that do not develop clinically overt bone metastases^55^. Interestingly, the bone metastatic niche was suggested to induce mesenchymal-epithelial transition (MET) in bone-disseminated breast cancer cells, thus supporting their metastatic outgrowth^56^.

We further analyzed the differences in gene expression between the parental cells and 4T-Bone, and found that bone-tropic tumor cells upregulated a gene signature related to physiologic bone functions, bone mineralization and remodeling. This phenomenon, termed “osteomimicry”, where breast cancer cells express proteins that are normally expressed in the bone microenvironment (e.g. by osteoblasts), and are linked to physiologic bone functions, was previously implicated in facilitating bone tropism and growth in bones^41,42^. Moreover, ectopic instigation of epithelium-to-osteomimicry transition in breast cancer cells enhanced bone metastasis in mouse models of breast cancer^57^. Interestingly, primary breast tumors that display micro-calcifications detected by mammographic screening are associated with decreased survival and increased risk of bone metastasis^58^, suggesting that osteomimicry may be a feature of tumor cell subpopulations in the primary tumor that provides them with bone-metastatic advantages. Taken together, our results suggest that acquisition of EMT and osteomimicry programs, combined with instigation of immunosuppression may endow breast cancer cells with bone metastatic capacity.

In summary, we established a bone-seeking variant of breast cancer cells that spontaneously forms aggressive bone metastases following surgical resection of a primary tumor. Characterization of the transcriptional changes and modifications in the immune microenvironment in the bone metastatic niche indicated that acquiring mesenchymal and osteomimetic features, combined with immunosuppression, are features of bone metastatic fitness. This model provides a clinically relevant platform to study the functional interactions between breast cancer cells and the bone microenvironment, in an effort to identify novel targets for intervention.

## Methods

All methods were carried out in accordance with relevant guidelines and regulations.

### Mouse strains

All animals were maintained within the Tel Aviv University Specific Pathogen Free (SPF) Facility. All Animal procedures included in the study were granted ethical approval by the Tel Aviv University Institutional Animal Care and Use Committee. BALB/c mice were purchased from Envigo, Israel. Mice were used for experiments at 6–9 weeks of age.

### Cell lines

The 4T1 cell line was purchased from the ATCC. All cell lines were routinely tested for mycoplasma using the EZ-PCR-Mycoplasma test kit (Biological Industries; 20-700-20). The cell lines were not authenticated.

### Spontaneous bone-tropic cell line generation

To generate a bone tropic variant of 4T1, cells were isolated from spontaneous bone metastases: Following orthotopic injection and primary tumor excision, mice were monitored for signs of morbidity/relapse every other day. Bones were then flushed in order to isolate cancer cells which were cultured thereafter. Cells were cleaned from possible immune/endothelial cell contamination by FACS sorting for EpCAM^+^CD45^−^CD31^−^, and reinjected orthotopically for another cycle of in vivo selection. Antibodies used: CD31(PECAM-1)-FITC (eBioscience, 11-0311-82, diluted 1:50), CD45-PE/Cy7 (eBioscience, 25-0451-82, diluted 1:200), and EpCAM-APC (eBioscience, 17-5791-80, diluted 1:100). Cells were designated 4T1.1 (first cycle) and 4T-Bone (second cycle). Sorting was performed using BD FACSAria II.

### Orthotopic tumors

4T1, 4T1.1 or 4T-Bone cells were injected. A total of 0.5 × 10^6^ cells were re-suspended in PBS and mixed 1:1 with Matrigel (356,231, BD Biosciences) to a final volume of 100μL. Mice were anesthetized by Ketamine (100 mg/kg) and Xylazine (10 mg/kg) solution (1:1), and cells were inoculated into the fourth mammary fat pad. Tumors were measured every other day using calipers, and tumor volumes were calculated using the formula X^2^ × Y × 0.5 (X-smaller diameter, Y-larger diameter). Tumors were excised ~ 16 days later, following anesthesia with Ketamine and Xylazine solution. Tissues were embedded in OCT or digested into single cell suspensions for further analysis.

### Detection of bone macrometastases

Mice were routinely checked for morbidity (e.g. paraplegia or difficulty of movement). Macro-metastatic bone lesions were detected by intra-vital imaging (μCT). Mice were euthanized at day 40, and bones of the legs, sternum, ribs and spinal column were taken to histopathological analysis in order to verify metastases.

### Bone histology

#### Bone fixation and decalcification

Bones were harvested from mice and fixated in 4% paraformaldehyde for 24 h. Fixated bones were then decalcified using EDTA solution at 37 °C for additional 18 h. Decalcified bones were embedded in paraffin.

#### H&E quantification

Bone tissue sections were stained with H&E using Sakura Tissue-Tek Prisma (Department of Pathology, Tel Aviv Sourasky Medical Center). Bone metastasis incidence was analyzed by a pathologist (Dr. Ibrahim Fahoum). Quantification of bone metastatic load was performed by analyzing the number of metastatic lesions per section or by evaluating the metastatic area per section using ImageScope.

### RNA isolation and Quantitative real-time PCR

RNA was isolated and qRT-PCR was performed as previously described^59^. Briefly, primary tumors were enzymatically digested to prepare single cell suspension. Single cells were collected into TRIzol LS reagent (Life Technologies; 10296-028) and RNA was isolated according to the manufacturer’s instructions. Alternatively, for in vitro experiments, total RNA was isolated from cell pellets or cell monolayers using the PureLink RNA Mini Kit (Invitrogen; 12183018A). RNA concentration and purity were analyzed using NanoDrop 2000c Spectrophotometer. cDNA synthesis was conducted using qScript cDNA Synthesis Kit (Quanta Biosciences, 95047-025).

RNA from FACS sorted granulocytes and T using was purified using EZ-RNA II Total RNA Isolation Kit (20-410-100; Biological Industries). Quantitative real-time PCRs (qRT-PCR) for mouse genes were conducted using SYBR Green FastMIX (Quanta Biosciences, P/N84071) in a StepOne Real-Time PCR System. All experiments were performed in triplicate. RQ (2^−ΔCt^) was calculated. Relative expression was normalized to GAPDH, UBC and GUS. All primers and oligonucleotide sequences used are shown in Table 1.

**Table 1:** Primer sequences.

Gene symbol	Forward sequence	Reverse sequence
Cdh1	ACACCGTAGTCAACGATCCTGA	GCCTCAAAATCCAAGCCCTT
Krt19	GAGGACTTGCGCGACAAGAT	CGTGTTCTGTCTCAAACTTGGTTCT
SNAI1	CACACGCTGCCTTGTGTCT	GGTCAGCAAAAGCACGGTT
vimentin	TTTCTTCCCTGAACCTGAGAGAA	GTCCATCTCTGGTCTCAACCGT
TWIST1	CGGAGACCTAGATGTCATTGTTT	CGCCCTGATTCTTGTGAATTTG
Tgfb1	CTGAACCAAGGAGACGGAATAC	GGGCTGATCCCGTTGATTT
Alpl	GGAATACGAACTGGATGAGAAGG	GGTTCCAGACATAGTGGGAATG
lbsp	CGGCCACGCTACTTTCTTA	GAACTATCGCCGTCTCCATTT
BGLAP	AGGGCAGCACAGGTCCTAA	CCAAGCAGGAGGGCAATA
RUNX2	CCACAAGGACAGAGTCAGATTACA	TGGCTCAGATAGGAGGGGTA
Osx	GGAGACCTTGCTCGTAGA	CAGAGAGACACCCACAGA
GAPDH	TGTGTCCGTCGTGGATCTGA	TTGCTGTTGAAGTCGCAGGAG
UBC	GTTACCACCAAGAAGGTC	GGGAATGCAAGAACTTTATTC
GUS	GCAGCCGCTACGGGAGTC	TTCATACCACACCCAGCCAAT

### FACS analysis and sorting

Bone marrow, spleen, lungs or blood cells were isolated from mice. Single cell suspensions were prepared according to organ (BM was flushed out, lungs and primary tumors were enzymatically digested, spleens were mechanistically digested). Single cell suspensions filtered by 70 μM cell strainers (Corning); red blood cells were lysed. Cells were counted and resuspended in FACS buffer (PBS with 2% FCS and 2 mM EDTA). Cells were then incubated for 15 min with anti-mouse CD16/CD32. Immune cell infiltration was analyzed by staining with the following anti-mouse antibodies: CD45 (BLG-103151), CD11b (BLG-101216), CD11c (eBioscience, 45-0114), SiglecF (BD565183), Ly6G (BLG-127614), Ly6C (BLG-128006), NKp46 (BLG-137618), B220 (BLG-103236), CD4 (BLG-100413), CD8a (BLG-100712), CD3 (eBioscience, 11-0031). DAPI was used to exclude dead cells (Molecular probes, Eugene, OR, USA; D3571). Analysis was performed with CytoFLEX Flow Cytometer (Beckman Coulter, Inc.) and data analysis was done with FlowJo Software (version X.0.7).

#### FACS sorting of Granulocytes and T cells

Sorting was performed using BD FACS Aria III. Granulocytes were isolated as CD45^+^CD11b^+^Ly6C^int^Ly6G^+^. T cells were isolated as CD45^+^CD3^+^ cells.

### Arginase activity assay

Arginase activity was assessed using QuantiChrom Arginase Assay Kit (DARG-100, BioAssay Systems) according to manufacturer’s instruction. Briefly, 8X10^5^ CD45^+^CD11b^+^Ly6C^int^Ly6G^+^ cells were harvested from BM and spleens, cells were washed in PBS and centrifuged at 1000 g for 10 min. Cell pellets were lysed in 100μL of 10 mM Tris–HCl (pH 7.4) containing 1 μM pepstatin A, 1 μM leupeptin, and 0.4% (w/v) Triton X-100, followed by 10 min centrifuge at 14,000 g. Supernatant was used for arginase assay.

### Immunofluorescence staining of primary tumor sections

#### Mouse samples tissue preparation

Mammary tumors were shortly washed in PBS. Fresh-frozen tissues were embedded in optimal cutting temperature compound (OCT; Tissue-Tek) on dry ice. Serial sections were obtained to ensure equal sampling of the examined specimens (10 μm trimming).

#### Immunofluorescence

Tissue sections were incubated overnight at 4 °C with the following anti-mouse antibodies: E-cadherin (Cell signaling; 24E10), αSMA (Sigma-Aldrich; F3777), Vimentin (Millipore; AB1620), N-cadherin (Cell signaling; D4R1H), Wide spectrum cytokeratin (Abcam; ab9377). Fluorescently conjugated secondary antibodies (Jackson ImmunoResearch Laboratories): Rhodamine Red-X–conjugated donkey anti-rabbit (711-295-152), Rhodamine Red-X–conjugated donkey anti-goat (705-295-147), Alexa Fluor 488–conjugated donkey anti-goat (705-545-147), and Alexa Fluor 488–conjugated goat anti-rabbit (111-545-144) were applied for 2 h at room temperature. Sections were then washed, incubated with DAPI (1:2000, Molecular Probes; D3571) and mounted with VECTASHIELD HardSet antifade mounting medium (VE-H-1400, Vector Laboratories). Slides were visualized and analyzed using confocal microscopes Leica SP5 or SP8s with a 63×/1.4 oil objective. Quantitative analyses were performed using ImageJ Software.

### Statistical analyses

Statistical analyses were performed using GraphPad Prism software as previously described^60^. Briefly, for two groups, statistical significance was calculated using t-test with Welch correction unless otherwise stated. For more than two comparisons, One-Way ANOVA with Tukey correction for multiple comparisons was applied unless otherwise stated. All tests were two-tailed. *p* value of ≤ 0.05 was considered statistically significant unless otherwise stated. Bar graphs represent mean and SD or mean and SEM across experimental repeats, as stated. All experiments were repeated at least three times.

## Supplementary information

Supplementary Figure 1.
