# Supplementary material for: Bone metastasis is associated with acquisition of mesenchymal phenotype and immune suppression in a model of spontaneous breast cancer metastasis
Source: Sci Rep. 2020 Aug 14;10:13838. doi: 10.1038/s41598-020-70788-3 (PMC7429866; doi:10.1038/s41598-020-70788-3)
Supplement: Supplementary file 1 — Supplementary Figure 1. [file 41598_2020_70788_MOESM1_ESM.pdf]

Supplementary information:

Bone metastasis is associated with acquisition of mesenchymal phenotype and immune suppression in a model of spontaneous breast cancer metastasis

Lea Monteran[1], Nour Ershaid[1], Idan Sabah[1], Ibrahim Fahoum[2], Yael Zait[1], Ophir Shani[1], Noam Cohen[1], Anat Eldar-Boock[3], Ronit Satchi-Fainaro[3] and Neta Erez[1*]

[1]Department of Pathology, Sackler Faculty of Medicine, Tel Aviv University, Tel Aviv 69978, Israel
[2]Department of Pathology, Tel Aviv Sourasky Medical Center, Tel Aviv University, Tel Aviv, Israel.
[3]Department of Physiology and Pharmacology, Sackler Faculty of Medicine, Tel Aviv University, Tel Aviv 69978, Israel

[*]Correspondence to netaerez@tauex.tau.ac.il

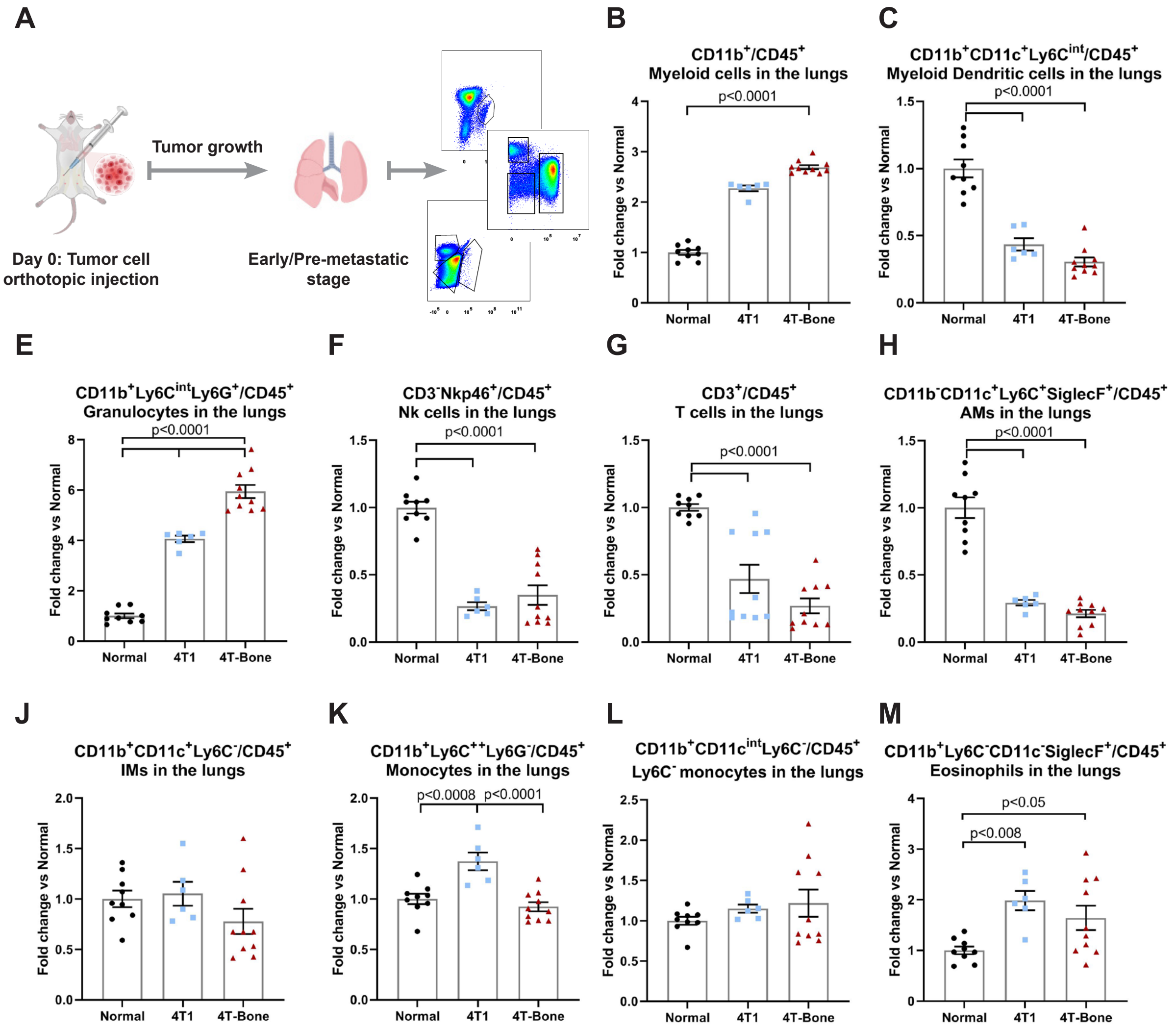

Supplementary Figure 1: Breast cancer metastasis is associated with changes in the immune milieu in lungs. (A) Scheme of experimental design. This figure was designed by using graphical elements from BioRender. (B-M) FACS analysis quantification of major immune cell populations in the lungs of normal, 4T1 and 4T-Bone-injected mice. (B) CD45^{+}CD11b^{+}CD11c^{-} total myeloid cell population. (C) CD45^{+}CD11b^{+}CD11c^{+}Ly6C^{int} myeloid-dendritic cells. (D) CD45^{+}CD11c^{+}CD11b^{-} dendritic cells. (E) CD45^{+}CD11c^{-}CD11b^{+}Ly6G^{+}Ly6C^{int} granulocytes. (F) CD45^{+}B220^{-}CD3^{-}Nkp46^{+} NK cells. (G) CD45^{+}B220^{-}CD3^{+} T cells. (H) CD45^{+}CD11c^{+}CD11b^{-}SiglecF^{+}Ly6C^{+} Alveolar Macrophages (AMs). (I) CD45^{+}B220^{+}CD3^{-} B cells. (J) CD45^{+}CD11c^{+}CD11b^{+}Ly6C^{-} interstitial macrophages (IMs). (K) CD45^{+}CD11c^{-}CD11b^{+}Ly6G^{-} Ly6C^{+} monocytes. (L) CD45^{+}CD11b^{+}CD11c^{int}Ly6C^{-}Ly6C^{-} monocytes. (M) CD45^{+}CD11c^{-}CD11b^{+}SiglecF^{+}Ly6G^{-} eosinophils. n=6-10 mice per group. Data are all presented as % of CD45, normalized to the average of normal group. Error bars represent SEM. $p<0.05$; Kruskal-Wallis Multiple comparison test.
